# A novel cell-penetrating peptide suppresses breast tumorigenesis by inhibiting β-catenin/LEF-1 signaling

**DOI:** 10.1038/srep19156

**Published:** 2016-01-11

**Authors:** Tsung-Hua Hsieh, Chia-Yi Hsu, Cheng-Fang Tsai, Chien-Chih Chiu, Shih-Shin Liang, Tsu-Nai Wang, Po-Lin Kuo, Cheng-Yu Long, Eing-Mei Tsai

**Affiliations:** 1 Department of Obstetrics and Gynecology, Kaohsiung Medical University Hospital, Kaohsiung Medical University, Kaohsiung, Taiwan; 2 Research Center for Environmental Medicine, Kaohsiung Medical University, Kaohsiung, Taiwan; 3 Center for Stem Cell Research, Kaohsiung Medical University, Kaohsiung, Taiwan; 4 Center for Infectious Disease and Cancer Research, Kaohsiung Medical University, Kaohsiung, Taiwan; 5 Graduate Institute of Medicine, College of Medicine, Kaohsiung Medical University, Kaohsiung, Taiwan

## Abstract

The inhibition of *β*-catenin/LEF-1 signaling is an emerging strategy in cancer therapy. However, clinical targeted treatment of the *β*-catenin/LEF-1 complex remains relatively ineffective. Therefore, development of specific molecular targets is a key approach for identifying new cancer therapeutics. Thus, we attempted to synthesize a peptide (TAT-NLS-BLBD-6) that could interfere with the interaction of β-catenin and LEF-1 at nuclei in human breast cancer cells. TAT-NLS-BLBD-6 directly interacted with β-catenin and inhibited breast cancer cell growth, invasion, migration, and colony formation as well as increased arrest of sub-G1 phase and apoptosis; it also suppressed breast tumor growth in nude mouse and zebrafish xenotransplantation models, showed no signs of toxicity, and did not affect body weight. Furthermore, the human global gene expression profiles and Ingenuity Pathway Analysis software showed that the TAT-NLS-BLBD-6 downstream target genes were associated with the HER-2 and IL-9 signaling pathways. TAT-NLS-BLBD-6 commonly down-regulated 27 candidate genes in MCF-7 and MDA-MB-231 cells, which are concurrent with Wnt downstream target genes in human breast cancer. Our study suggests that TAT-NLS-BLBD-6 is a promising drug candidate for the development of effective therapeutics specific for Wnt*/β*-catenin signaling inhibition.

Breast cancer is a common malignancy and ranks as the fourth leading cause of cancer mortality in women worldwide^1^. Early study revealed that mutation of mammary ducts and lobules is the main contributory factor. There are many factors that affect mutation including genetics^2^, lifestyle^3^, viral infections^4^, tobacco use^5^, and environment^6^. In current therapy, breast cancer patients are mainly treated with surgery, chemotherapy, and a combination regimen^7^. Interestingly, new drug development has been enhanced by advancements in targeted gene therapy^8^. Targeted gene therapy includes the successful application of monoclonal antibodies (Her2/neu)^9^ and small peptides. The lack of toxicity and immunogenicity as well as stable cell penetration make small peptides a promising avenue for therapeutic development^10^. Among these targets, catenin beta-1/lymphoid enhancer factor-1 (*β*-catenin/LEF-1) is a very important complex in new drug development for small peptides.

The *β*-catenin**/**LEF-1 complex is a nuclear response transcription factor and plays an important role in the Wnt/wingless signaling pathway^11^. The Wnt/wingless pathway regulates many tumorigenic processes including cell growth^12^, invasion^13^, migration^14^, colony formation, and xenografting in human cancer^15^. When the Wnt receptor complex is unbound, β-catenin forms a complex with adenomatous polyposis coli (APC), axin, and glycogen synthase kinase 3b (GSK3b), which then undergoes ubiquitin-mediated degradation in the cytoplasm. Otherwise, upon Wnt binding to the receptor complex, β-catenin translocates to the nucleus and binds with the LEF-1/TCF co-transfection factor. It has been reported that the first 76 amino acids of LEF-1 are sufficient for the interaction with β-catenin^11^. In addition, early study found that β-catenin activates downstream target genes including BMP4^16^, MYC^17^, and cyclin D1^18^, which play important oncogenic roles to promote breast cancer migration, invasion, and tumorigenesis. Therefore, suppressing the *β*-catenin**/**LEF-1 signaling pathway is an important direction in new drug development.

In the current study, we synthesized a small peptide to block the interaction between β-catenin and LEF-1. The effects on tumorigenesis and the downstream target gene profile in human breast cancer were investigated *in vitro* and *in vivo*. This novel small peptide may be a candidate in future approaches for human breast cancer therapy.

## Results

### TAT-NLS-BLBD-6 inhibits the growth of breast cancer cells

**Figure 1:**
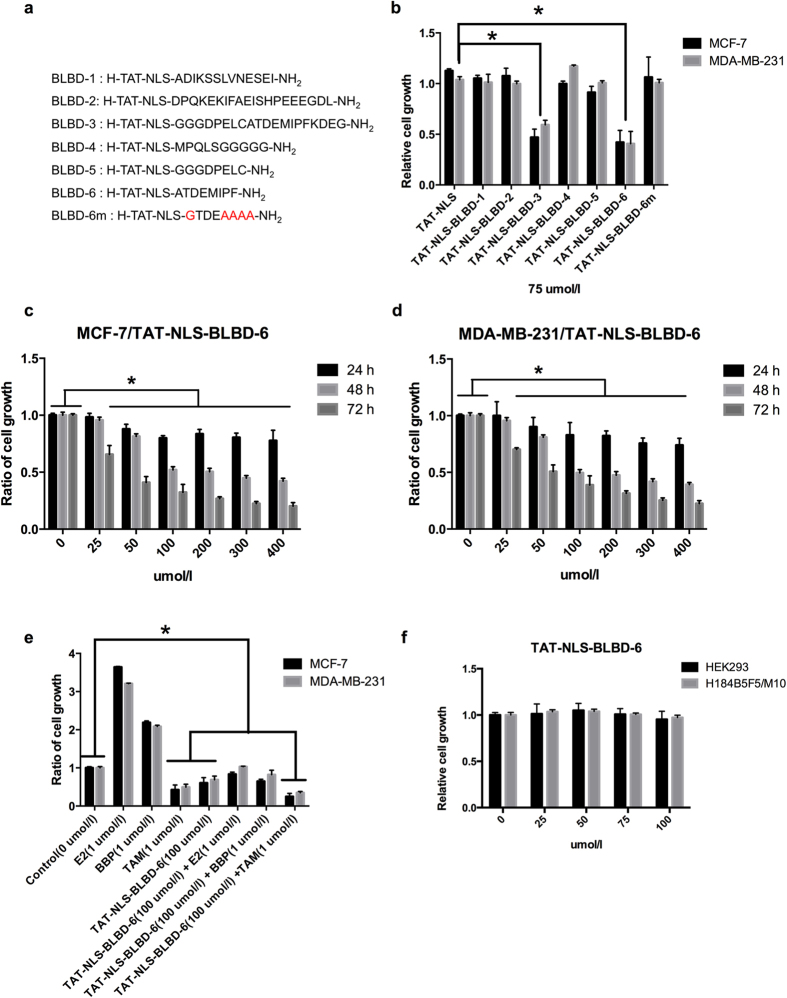
The effect of different BLBD fragments on growth of human breast cancer cells. (**a**) The seven peptides sequences used in this study. TAT and NLS were linked to six fragments of the β-catenin binding site on LEF-1 (TAT-NLS-BLBD-1, -2, -3, -4, -5, and -6) (see Supplementary Figure S1 online). TAT-NLS-BLBD-6m was generated by mutating the TAT-NLS-BLBD-6 sequence (red residues). (**b**) MCF-7 and MDA-MB-231 cells were transfected with 75 μmol/l TAT-NLS-BLBD fragment peptide. (**c,d**) Cells were transfected with the indicated concentrations of TAT-NLS-BLBD-6 for 24, 48, and 72 hr. (**e**) Cells were treated with E2 (1 μM), BBP (1 μM), TAM (1 μM), or a combination of TAT-NLS-BLBD-6 (100 μmol/l) and E2, BBP, or TAM, and the cell growth was evaluated after 48 hr. (**f**) H184B5F5/M10 and HEK293 cells were transfected with the indicated doses of TAT-NLS-BLBD-6 peptide, and cell growth was analyzed by CCK-8 at 48 hr post-transfection. Data are the means ± SD of three experiments. *P < 0.05 *vs*. untreated control; two-tailed Student’s *t* test. Scare bare = 200 uM.

First, we synthesized successive short peptides of the *β*-catenin/LEF-1 binding domain^11^ (BLBD), transactivator of transcription (TAT, YGRKKRRQRRR), and nuclear localization signal (NLS, RKRRK) protein to form a fusion peptide (Fig. 1a). BLBD peptides were derived from the first 76 amino acids of LEF-1, which are sufficient for the interaction with β-catenin. TAT is a cell-penetrating peptide from the human immunodeficiency virus, and it can deliver proteins, DNA, RNA, and nanoparticles into the cytoplasm in a short time with extremely high efficiency^19^,^20^. However, because stabilized β-catenin translocates into the nucleus to affect TCF-4/LEF-1 binding to Wnt target genes^21^, we synthesized TAT-NLS fusion peptides derived from LEF-1 to analyze their effects on β-catenin-mediated signaling in the nuclei of breast cancer cells. We chemically synthesized six peptides with a variable region of the *β*-catenin/LEF-1 binding domain and one mutated version of BLBD-6 to find the capacity of these peptides to suppress cancer cell growth (Fig. 1a). A growth assay was used to screen the small peptides for their ability to suppress cancer cell growth. The results indicated that two synthetic peptides (TAT-NLS-BLBD-3 and -6) inhibited the growth of MCF-7 and MDA-MB-231 cells (Fig. 1b; also see Supplementary Fig. S1 online). BLBD-3 and BLBD-6 share a common sequence in the activation region of the *β*-catenin/LEF-1 binding domain (ATDEMIPF); thus, we mutated the activation region (GTDEAAAA, TAT-NLS-BLBD-6m) and found that TAT-NLS-BLBD-6m did not affect the growth of breast cancer cells compared with TAT-NLS-BLBD-6 (Fig. 1b). Therefore, the activation region sequence (ATDEMIPF) is responsible for the growth inhibition of human breast cancer cells.

Next, we analyzed the time and dose dependence of TAT-NLS-BLBD-6 on cell growth. The results indicated that TAT-NLS-BLBD-6 inhibited breast cancer cell growth in a time- and dose-dependent manner (Fig. 1c,d). We also examined the effect of combining BLBD-6 with drugs including E2 (1 μM), BBP (1 μM), and TAM (1 μM) with BLBD-6 in breast cancer cells. TAT-NLS-BLBD-6 blocked the activity of E2 and BBP, but increased the response of TAM in breast cancer cells (Fig. 1e). Interestingly, TAT-NLS-BLBD-6 did not inhibit the growth of human normal mammary epithelial cell H184B5F5/M10 and embryonic kidney 293 (HEK293) cells (Fig. 1f). Together, these results suggested that the ATDEMIPF sequence of TAT-NLS-BLBD-6 inhibits growth of breast cancer cells, but not of normal cells such as H184B5F5/M10 and HEK293.

### TAT-NLS-BLBD-6 specifically binds to β-catenin in the nucleus

**Figure 2:**
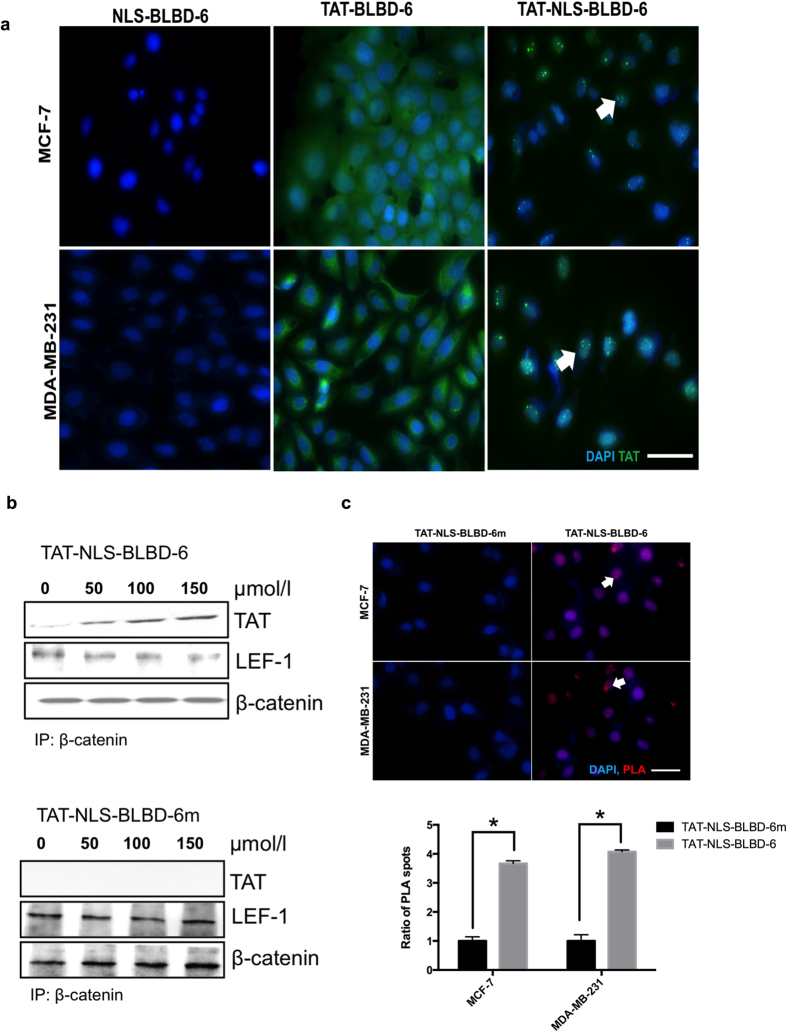
The subcellular distribution and specific binding of TAT-NLS-BLBD-6 in human breast cancer cells. (**a**) MCF-7 and MDA-MB-231 cells were transfected with 100 μmol/l NLS-BLBD-6, TAT-BLBD-6, or TAT-NLS-BLBD-6 for 2 hr before washing and fixing. The anti-TAT primary antibody and Alexa-488 secondary antibody were stained by immunofluorescence, and the fluorescence image was captured by microscopy. Arrows show the TAT-NLS-BLBD-6 peptide distribution in the nucleolus. (**b**) MCF-7 cells were transfected with the indicated concentrations of TAT-NLS-BLBD-6 and TAT-NLS-BLBD-6m. The protein–protein interaction between TAT and β-catenin or LEF-1 and β-catenin was evaluated by immunoprecipitation with anti-β-catenin and immunoblotting with the indicated antibodies at 2 hr post-transfection. (**c**) MCF-7 and MDA-MB-231 cells were transfected with 100 μmol/l TAT-NLS-BLBD-6 or TAT-NLS-BLBD-6m and the protein–protein interaction between TAT and β-catenin was evaluated by immunofluorescence with the PLA at 2 hr post-transfection. Arrows show the protein–protein interaction in the nucleolus.

Although TAT-NLS-BLBD-6 inhibited the growth of breast cancer cells, it was not clear whether TAT-NLS-BLBD-6 could enter into the nucleus and bind the *β*-catenin elements *in vitro*. We first determined the subcellular distribution of TAT-NLS-BLBD-6 peptide in the cell. Immunofluorescence staining indicated that TAT-NLS-BLBD-6 (100 μmol/l) was located in the nuclei in both breast cancer cell lines (Fig. 2a). Next, we analyzed the peptide–protein interaction by immunoprecipitation and PLA assay. These assays indicated that TAT-NLS-BLBD-6 bound to the β-catenin elements in MCF-7 and MDA-MB-231 cell nuclei (Fig. 2b,c).

### TAT-NLS-BLBD-6 induces apoptosis and inhibits invasion, migration, and colony formation

**Figure 3:**
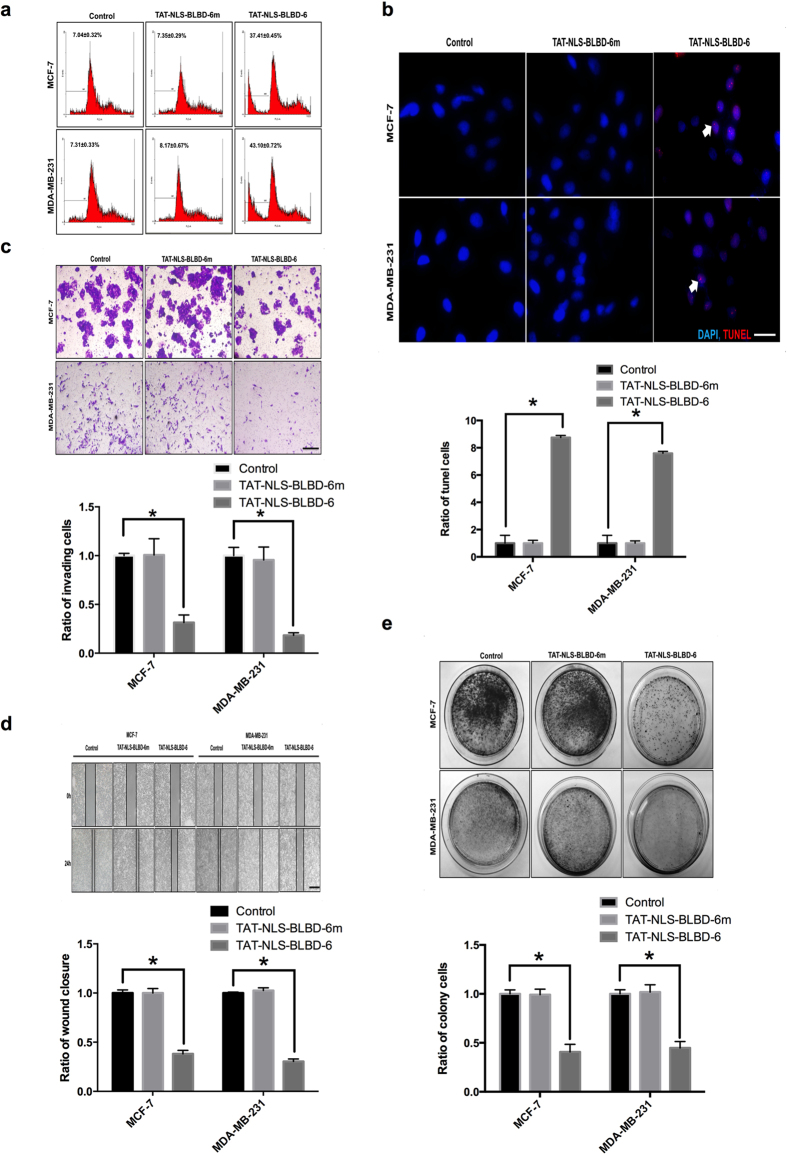
The biological function of TAT-NLS-BLBD-6 *in vitro*. MCF-7 and MDA-MB-231 cells were transfected with control, 100 μmol/l TAT-NLS-BLBD-6 or TAT-NLS-BLBD-6m. (**a**) Cell cycle progression was analyzed by propidium iodide staining and flow cytometry. (**b**) Apoptosis was analyzed by the TUNEL assay. Arrows show apoptosis in the nucleolus. Motility was analyzed by the invasion assay (**c**) and wound-healing assay (**d**) at 48 hr post-transfection. Data are the means ± SD of three experiments. *P < 0.05 *vs*. untreated control; two-tailed Student’s *t* test. (**e**) Cell proliferation was analyzed by the colony-formation assay at 14 day post-transfection. Scare bare = 200 uM.

To further investigate the biological effects of these peptides, we evaluated apoptosis, invasion, migration and colony formation. We used flow cytometry and the TUNEL assay to examine the effects of control, TAT-NLS-BLBD-6 and TAT-NLS-BLBD-6m apoptosis. Flow cytometry showed that, compared with control and TAT-NLS-BLBD-6m, TAT-NLS-BLBD-6 increased the sub-G1 phase region (pro-apoptotic effect) from 7.35% to 37.41% in MCF-7 and from 18.34% to 43.10% in MDA-MB-231 (Fig. 3a). The TUNEL assay showed that TAT-NLS-BLBD-6 also increased the effect of binding with BrdU and DNA fragmentation when compared with control or TAT-NLS-BLBD-6m in breast cancer cells (Fig. 3b). In addition, invasion, migration and colony-formation assays indicated that TAT-NLS-BLBD-6 inhibited the mobility and proliferation of breast cancer cells when compared with control or TAT-NLS-BLBD-6m (Fig. 3c–e). We used normal cell lines to analyze the phenotypic features of TAT-NLS-BLBD-6. The results revealed that TAT-NLS-BLBD-6 had no effect on the phenotypic features including cell cycles, apoptosis, invasion, migration and colony formation assay in normal cell lines H184B5F5/M10 (Supplementary Fig. S2 online). Therefore, TAT-NLS-BLBD-6 has the potential to mediate biological function in human breast cancer cells.

### TAT-NLS-BLBD-6 inhibits tumor growth in the xenograft and xenotransplantation models

**Figure 4:**
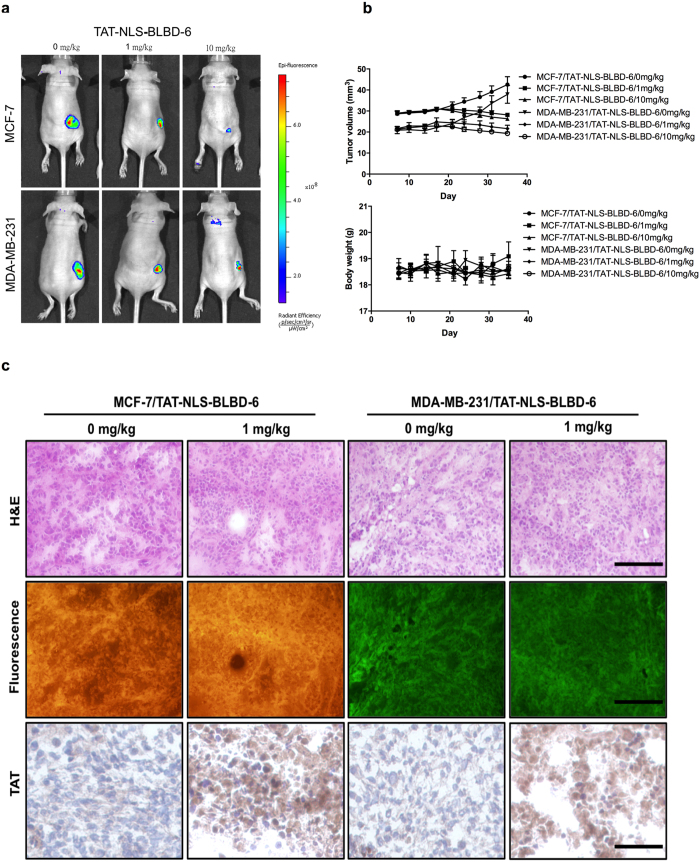
TAT-NLS-BLBD-6 inhibits tumor growth in nude mice. MCF-7-GFP or MDA-MB-231-GFP cells were injected into the right side flanks of SCID nude mice (n = 5 per group). The low dose (1 mg/kg) and high dose (10 mg/kg) of TAT-NLS-BLBD-6 were injected into the tumor once every 2 days for 35 days. (**a**) Tumor GFP images were captured by the IVIS system. (**b**) The tumor volumes and body weights of nude mice were calculated and recorded. (**c**) The solid tumor was cut at a thickness of 5 μm and examined using hematoxylin and eosin (H&E), fluorescence, and immunohistochemistry for TAT staining. Scare bare = 100 uM.

To evaluate the effects of the TAT-NLS-BLBD-6 peptides *in vivo*, we established a xenograft model in nude mice and a zebrafish xenotransplantation model. For the xenograft model, 1 × 10^7^ MCF-7-YFP and MDA-MB-231-GFP cells were subcutaneously injected into the right flanks of nude mice. After 1 week of implantation, the mice were treated with the control, TAT-NLS-BLBD-6m (1 mg/kg) and TAT-NLS-BLBD-6 (1 and 10 mg/kg) peptide through intratumoral injection, and the fluorescence density was analyzed by *In Vivo* Imaging System (IVIS) 35 days after inoculation. TAT-NLS-BLBD-6 inhibited tumor growth without having any effect on body weight when compared with the control peptide (Fig. 4a,b, also see Supplementary Fig. S3 online). In addition, we obtained tumor sections and confirmed that they originated from the injected breast cancer cells, which were positive for YFP or GFP. Immunohistochemistry staining revealed that TAT expression was high and located in the nuclei in the tumors injected with TAT-NLS-BLBD-6 compared with those injected with control peptide (Fig. 4c).

**Figure 5:**
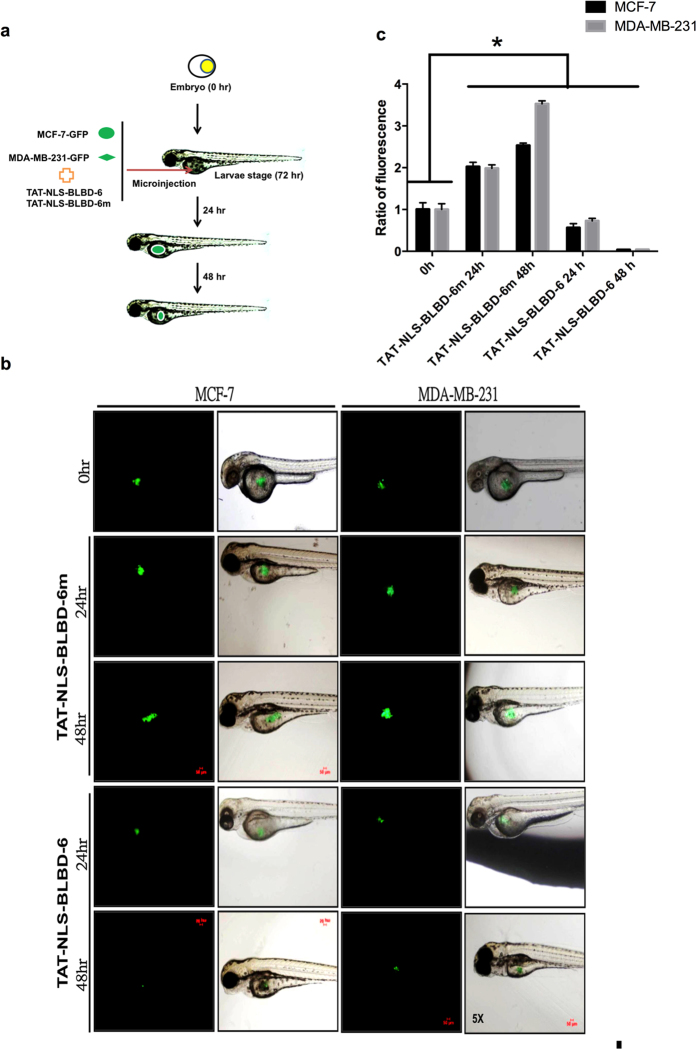
TAT-NLS-BLBD-6 inhibits tumor growth in zebrafish. (**a,b**) MCF-7-GFP or MDA-MB-231-GFP cells and TAT-NLS-BLBD-6 were microinjected into the zebrafish embryos (larvae stage, n = 20 per group). Fluorescence imaging of the whole body of the zebrafish was performed by microscopy 24 hr and 48 hr after transplantation. (**c**) The photon flux intensity was quantitated by MetaMorph software.

To examine zebrafish xenotransplantation, 1 × 10^4^ MCF-7-GFP and MDA-MB-231-GFP cells were co-injected with TAT-NLS-BLBD-6 or TAT-NLS-BLBD-6m peptide (100 μmol/l) into the yolk sacs of zebrafish embryos. Fluorescence density was captured by fluorescence microscopy at 0, 24 and 48 hr after implantation (Fig. 5a). The fluorescence density was gradually reduced between 24 hr and 48 hr in the TAT-NLS-BLBD-6 group compared with the TAT-NLS-BLBD-6m group (Fig. 5b,c). Thus, TAT-NLS-BLBD-6 might represent a potential therapeutic strategy to suppress breast tumor growth without toxicity for body weight.

### Downstream genes were consistently identified in the TAT-NLS-BLBD-6 and *β*-catenin/TCF4/LEF-1 complex

**Figure 6:**
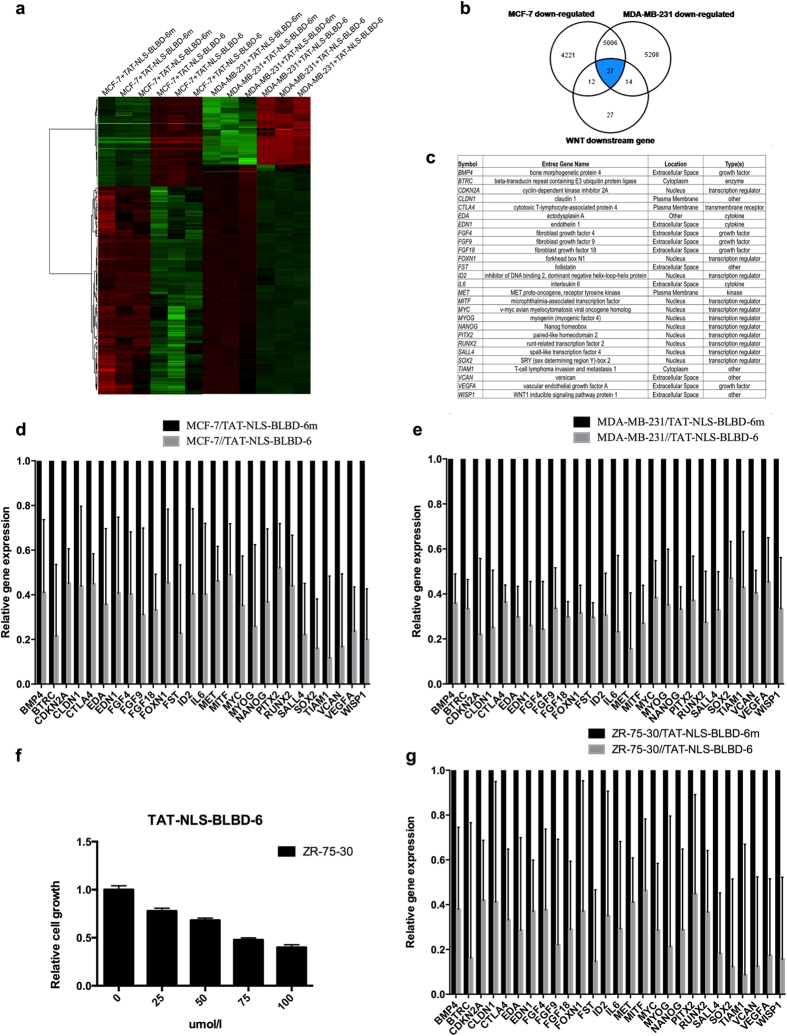
Identifying the downstream target genes of TAT-NLS-BLBD-6. (**a**) MCF-7 and MDA-MB-231 cells were transfected with 100 μmol/l TAT-NLS-BLBD-6 or TAT-NLS-BLBD-6m. The human global gene expression profiles of three independent RNA samples were analyzed by human oligonucleotide DNA microarray at 48 hr post-transfection. (**b,c**) Of the genes commonly down-regulated by TAT-NLS-BLBD-6 in MCF-7 and MDA-MB-231, 27 were identical to *β*-catenin/TCF4/LEF-1 downstream target genes. (**d,e,g**) Using Q-PCR, the 27 candidate genes in MCF-7, MDA-MB-231 and ZR-75-30 cells were examined at 48 hr post-transfection with 100 μmol/l TAT-NLS-BLBD-6 that the expression of each gene is shown relative to the expression in cells transfected with TAT-NLS-BLBD-6m. Data are the means ± SD of three experiments. (**f**) ZR-75-30 cells were transfected with control, 100 μmol/l TAT-NLS-BLBD-6 or TAT-NLS-BLBD-6m. Cell growth was analyzed by CCK-8 at 48 hr post-transfection. Data are the means ± SD of three experiments.

The above results indicated that TAT-NLS-BLBD-6 can bind to β-catenin. To determine whether it can affect the genes downstream of *β*-catenin/TCF4/LEF-1, the human global gene expression profiles of three independent RNA samples from the breast cancer cells treated with TAT-NLS-BLBD-6 and TAT-NLS-BLBD-6m were examined (Fig. 6a). The gene expression profile data were analyzed by Ingenuity Pathway Analysis (IPA) software. The results indicated that 27 genes that were down-regulated in both in MCF-7 and MDA-MB-231 cells were also identified as *β*-catenin/TCF4/LEF-1 downstream genes (Fig. 6b). These genes are *BMP4*, *BTRC*, *CDKN2A*, *CLDN1*, *CLTA4*, *EDA*, *EDN1*, *FGF4*, *FGF9*, *FGF18*, *FOXN1*, *FST*, *ID2*, *IL6*, *MET*, *MITF*, *MYC*, *MYOG*, *NANOG*, *RUNX2*, *PITX2*, *SALL4*, *SOX2*, *ITAM1*, *VCAN*, *VEGFA*, and *WISP1* (Fig. 6c). Next, we used Q-PCR to confirm the gene expression profile data in breast cancer cells. Indeed, the gene expression of the 27 candidate genes decreased following TAT-NLS-BLBD-6 treatment compared with TAT-NLS-BLBD-6m treatment in MCF-7 (Fig. 6d) and MDA-MB-231 (Fig. 6e) cells. Together, these findings suggest that TAT-NLS-BLBD-6 can inhibit the expression of *β*-catenin/TCF4/LEF downstream genes.

### The signaling pathways of down-regulated genes of TAT-NLS-BLBD-6

To further evaluate the pathway maps and molecular and cellular functions of the genes down-regulated by TAT-NLS-BLBD-6, IPA software was used to identify the top two pathways as IL-9 (*p* = 2.4E–02, 11.8%) and HER-2 signaling in breast cancer (*p* = 3.76E–02, 7.9%). Furthermore, the top five functions identified by IPA were lipid metabolism (*p* = 3.45E–05~4.84E–02), molecular transport (*p* = 3.45E–05~4.84E–02), small-molecule biochemistry (*p* = 3.45E–05~4.84E–02), cellular development (*p* = 1.35E–04~4.88E–02), and cellular growth and proliferation (*p* = 1.35E–04~4.81E–02). To further investigate whether TAT-NLS-BLBD-6 mediated HER-2 signaling pathway, we used one HER2 positive cell line ZR-75–30 to analyze the ability of growth and the gene expression of downstream genes when the cells were treated with TAT-NLS-BLBD-6. The results demonstrated that TAT-NLS-BLBD-6 inhibits the growth (Fig. 6f) and the expression of β-catenin/TCF4/LEF downstream genes (Fig. 6g) in human HER2 positive cell line ZR-75–30. Overall, the IPA software provided evidence that TAT-NLS-BLBD-6 may affect inflammatory response, cellular development, and growth and proliferation in human breast cancer (see Supplementary Fig. S4 online).

## Discussion

The Wnt/*β*-catenin signaling pathway regulates the transcription of many genes involved in tumorigenesis and is a potential therapeutic target in cancer progression. Previous reports have shown that Wnt inhibition is a potential therapeutic strategy and can suppress cell growth and invasion in human cancer^22^,^23^. In our current study, we synthesized a small peptide TAT-NLS-BLBD-6 (ATDEMIPF), which is a Wnt/*β*-catenin signaling inhibitor and prevents the binding of β-catenin to LEF-1 at the nuclear region of human breast cancer cells. TAT-NLS-BLBD-6 increased apoptosis and decreased cell growth and motility. Furthermore, we demonstrated that TAT-NLS-BLBD-6 inhibited expression of 27 *β*-catenin**/**LEF-1 downstream target genes, including *BMP4*, *BTRC*, *CDKN2A*, *CLDN1*, *CLTA4*, *EDA*, *EDN1*, *FGF4*, *FGF9*, *FGF18*, *FOXN1*, *FST*, *ID2*, *IL6*, *MET*, *MITF*, *MYC*, *MYOG*, *NANOG*, *RUNX2*, *PITX2*, *SALL4*, *SOX2*, *ITAM1*, *VCAN*, *VEGFA*, and *WISP1*. Among these downstream target genes, *CDKN2A*, *CLDN1*, *CLTA4*, *IL6*, *MYC*, *NANOG*, *SOX2*, *VEGFA*, and *WISP1* are known to be potential prognostic factors and have been considered to be oncogenes in various cancers^17^,^24^,^25^,^26^,^27^,^28^,^29^,^30^.

Various preclinical approaches have been used to inhibit Wnt/*β*-catenin signaling pathways in cancer progression. These modalities, including protein depletion by a neutralizing anti-Dickkopf-1 (DKK1) antibody^31^, the soluble antagonist secreted Frizzled-related protein 2 (sFRP-2)^15^,^32^, and disheveled PDZ peptides^33^, inhibit tumor growth. Our results found that TAT-NLS-BLBD-6 inhibited cancer cell growth, invasion, and migration *in vivo* and *in vitro*, but not in normal cells. Therefore, we believe that using TAT-NLS-BLBD-6 (ATDEMIPF) peptide may be an effective therapeutic approach for human breast cancer without harm to normal cells. Consistent with our finding regarding the role of the interaction binding sequence, A_17_TDEMIPF_24_, structure analysis found that the LEF-1 residues responsible for binding to β-catenin are Asp19, Met21, Ile22, and Phe24^34^. The ATDEMIPF had the greatest effect on progression of human breast cancer compared with other sequences.

Previous investigations have reported that environmental hormone factors, such as phthalates (e.g., BBP), stimulate breast cancer through activation of the Wnt/*β*-catenin signaling pathway that contributes to tumorigenesis^35^ and epithelial-mesenchymal transition^36^. Phthalates are important in breast cancer progression; they can induce translocation of β-catenin into the nucleus and activate the response transcription factors LEF-1/TCF to induce downstream target gene expression. Therefore, developing new drugs to block Wnt/*β*-catenin signaling will inhibit cancer progression induced by phthalates. In the present study, we demonstrated that TAT-NLS-BLBD-6 combined with BBP blocked cell growth induced by BBP. This result suggests that TAT-NLS-BLBD-6 is an inhibitor for breast cancer progression induced by phthalates.

HER-2 is a tyrosine-protein kinase 2 of the ErbB family^37^. Early study found that the *β*-catenin/LEF-1 was activated by HER-2 to induce proliferation, invasion and migration and survival of cancer cells^38^,^39^. In addition, IL-9 is a cytokine produced by T-cells^40^, which was the down-regulated gene of Wnt^41^. Interesting, we results showed that the TAT-NLS-BLBD-6 downstream target genes were associated with IL-9 and HER-2 signaling pathways. This global gene profiles and IPA software results were confirmed with previous study and demonstrate that TAT-NLS-BLBD-6 is an inhibitor of *β*-catenin/LEF-1 signaling pathway.

In summary, our study found that TAT-NLS-BLBD-6, a cell-penetrating pentapeptide, blocks the interaction between β-catenin and LEF-1 and also efficiently attenuates tumorigenesis *in vitro* and *in vivo.* These results suggest that TAT-NLS-BLBD-6 is an effective Wnt signaling inhibitor and may be a potential therapeutic agent of human breast cancer.

## Materials and Methods

### Cell culture and peptide synthesis

MCF-7 and MDA-MB-231 cells were purchased from American Type Culture Collection and maintained in DMEM/F12 medium containing 10% fetal bovine serum and 5% penicillin-streptomycin-amphotericin (Life Technologies, Grand Island, NY). All cells were incubated at 37 °C and 5% CO_2_. The following peptides were synthesized by Kelowna International Scientific Inc. (Taipei, Taiwan): TAT-NLS-BLBD-1, H-TAT-NLS-ADIKSSLVNESEI-NH_2_; TAT-NLS-BLBD-2, H-TAT-NLS-DPQKEKIFAEISHPEEEGDL-NH_2;_ TAT-NLS-BLBD-3, H-TAT-NLS-GGGDPELCATDEMIPFKDEG-NH_2_; TAT-NLS-BLBD-4, H-TAT-NLS-MPQLSGGGGG-NH_2_; TAT-NLS-BLBD-5, H-TAT-NLS-GGGDPELC-NH_2_; TAT-NLS-BLBD-6, H-TAT-NLS-ATDEMIPF-NH_2_; BLBD-6m, H-TAT-NLS-GTDEAAAA-NH_2_; TAT-BLBD-6, H-TAT-ATDEMIPF-NH_2_; NLS-BLBD-6, H-NLS-ATDEMIPF-NH_2_.

### Cell growth

Cell growth was analyzed using 2-(2-methoxy-4-nitrophenyl)-3-(4-nitroph enyl)-5-(2,4-disulfophenyl)-2H-tetrazolium, monosodium salt, and the Cell Counting Kit-8 (CCK-8, Sigma). MCF-7, MDA-MB-231, and HEK293 cells were seeded in 96-well plates and incubated with peptide BLBD1-6, 17β-estradiol (E2, 1 μM), benzyl butyl phthalate (BBP, 1 μM), and tamoxifen (TAM, 1 μM). After culturing for another 48 hr, cell growth was analyzed by CCK-8 and the optical density was detected at 450 nm. The growth ratio was normalized to the cells without treatment.

### Immunoprecipitation and western blotting

Immunoprecipitation and western blotting were performed as described previously^42^,^43^. MCF-7 and MDA-MB-231 cells were harvested in 4 °C phosphate-buffered saline and cell pellets were lysed with RIPA lysis buffer (Millipore, Bedford, MA, USA) for 30 min on ice. Cell lysis supernatant liquid was obtained by centrifugation at 10,000 × *g* for 10 min, incubated with protein-G beads (Roche, Indianapolis, IN) and anti-β-catenin, and subjected to western blotting. For the western blotting assay, cellular extract proteins were separated by SDS-polyacrylamide gel (SDS-PAGE) and transferred to nitrocellulose membrane (Millipore) using a dry transfer apparatus (Bio-Rad). After blocking nonspecific binding with 5% milk buffer, the membrane was incubated with primary antibodies: anti-TAT (Santa Cruz Biotechnology, Santa Cruz, CA, USA), anti-LEF-1(Epitomics, Burlingame, CA, USA) and anti-β-catenin (Epitomics, Burlingame, CA, USA). The proteins were visualized using ECL (Amersham Pharmacia Biotech) and coupled using the Bio-Rad Chemiluminescent Detection System.

### Immunofluorescence, TUNEL staining, and proximity ligation assay (PLA)

MCF-7 and MDA-MB-231 cells were cultured in 35-mm plates with cover slides on the plate bottom and treated with NLS-BLBD-6, TAT-BLBD-6 and TAT-NLS-BLBD-6 peptide for 24 hr. Subsequently, cells were fixed with 4% paraformaldehyde and permeabilized with 0.2% Triton X-100 for 20 min at room temperature. For the immunofluorescence assay, the slides were incubated with primary antibodies, anti-β-catenin (Epitomics) and anti-TAT (Santa Cruz), for 6 hr, and Alexa-488 fluorescence secondary antibodies for 1 hr. DAPI (Sigma, St Louis, MO) was used to stain the nucleus for 1 min at room temperature. For the TUNEL assay, apoptosis was analyzed by the Apo-BrdU-red DNA Fragmentation Assay kit, according to the manufacturer’s protocol (BioVision, Mountain View, CA, USA). For the PLA, the protein–protein interaction assay was analyzed by Duolink^®^ using PLA^®^ Technology, according to the manufacturer’s protocol (Olink Bioscience, Uppsala, Sweden). Briefly, the slides were incubated with primary antibodies, anti-β-catenin (Epitomics) and anti-TAT (Santa Cruz), and secondary antibodies, Duolink PLA Rabbit MINUS and PLA Mouse PLUS proximity probes. Finally, the proximity ligation was performed by the Duolink detection reagent kit (Olink Bioscience). The immunofluorescence image and TUNEL staining was photographed by a microscope (IX-71, Olympus, Tokyo, Japan).

### Cell cycle analysis

MCF-7 and MDA-MB-231 cells were plated in 6-well plates for 24 hr, and the medium was replaced with fresh culture medium containing 100 μmol/l peptide. After incubation for 24 hr, the cells were harvested by trypsinization and then fixed with 70% 4 °C ethanol. Intracellular DNA was stained with 50 ng/ml propidium iodide in the dark for 30 min at room temperature, and the percentages of sub-G1 cells were determined by flow cytometry (BD LSRII analyzer; BD Biosciences).

### Invasion, migration, and colony-formation assays

Transwell chambers of 8-mm pore size were used in the *in vitro* invasion assay. MCF-7 and MDA-MB-231 cells were seeded onto the upper chamber and incubated with TAT-NLS-BLBD-6 and TAT-NLS-BLBD-6m peptide and 10% fetal bovine serum medium were added to the bottom chamber well. After 24 hr, cells invading the lower chamber were fixed with 4% paraformaldehyde solution and stained with 0.1% crystal violet for 30 min at 37 °C. Three random field images were obtained by microscopy, and the number of invading cells were counted. For the migration assay, MCF-7 and MDA-MB-231 cells were seeded onto 6-well plates. After 24 hr, an artificial wound was created using a 10-μl pipette tip, and the wound was incubated with TAT-NLS-BLBD-6 or TAT-NLS-BLBD-6m for 24 hr at 37 °C. The images of wound healing were captured by microscopy, and the wound healing distances were calculated by Image J (U.S. National Institutes of Health, Bethesda, MD, USA). For the colony formation assay, MCF-7 and MDA-MB-231 cells were seeded into 10-cm dishes with the medium containing TAT-NLS-BLBD-6 or TAT-NLS-BLBD-6m. After 2 weeks, cells were fixed with 4% paraformaldehyde solution and stained with 0.1% crystal violet for 30 min at 37 °C.

### Tumor growth analysis *in vivo*

All animal experiments were approved by the Kaohsiung Medical University Institutional Animal Care and Use Committee (IACUC Approval No: 101156) and we accordance with the approved guidelines. Female nude mice (4–5 weeks old) were obtained from the National Laboratory Animal Center (Taipei, Taiwan). Xenograft tumor models were established by subcutaneous injection of 1 × 10^7^ MCF-7-YFP or MDA-MB-231-GFP cells into the right flanks of mice. Mice with palpable tumors were randomly divided into three groups with five animals in each group. The mice were treated with control peptide, 0, 1 and 10 mg/kg TAT-NLS-BLBD-6 by intratumoral injection once every 2 days for 35 days. The fluorescence density was analyzed by an *In Vivo* Imaging System (IVIS) (Berthold Technologies, Bad Wildbad, Germany), and the tumor volumes (V) of nude mice were calculated by: V = length × diameter^2^ × 0.5. For zebrafish xenotransplantation, zebrafishes (*Danio rerio*) were maintained at 28 °C in an air incubator, and 1 × 10^4^ MCF-7-GFP or MDA-MB-231-GFP cells combined with TAT-NLS-BLBD-6 or control peptide were injected into the zebrafish embryos according to the previously described protocol^44^. The fluorescence density was measured 24 and 28 hr after injection using an epifluorescence microscope.

### Histologic study of the tumor

Tumor tissues were sectioned to a thickness of 5 μm and mounted on microscope slides. Tissue slides were stained with a Dako LSAB kit (Dako, Carpinteria, CA) according to the manufacturer’s protocol. The TAT antibody (Santa Cruz) was used for immunohistochemistry, and the nuclei were stained with hematoxylin and eosin. The fluorescence images were captured by a fluorescence microscope (IX-71, Olympus, Tokyo, Japan).

### Human oligonucleotide DNA microarray

Total RNA was extracted from cells using the Trizol reagent (Invitrogen, Carlsbad, CA, USA). The RNA concentration and purity were checked by OD_260_/OD_280_ (>1.8) and OD_260_/OD_230_ (>1.6), and the yield and quality were accessed using an Agilent 2100 Bioanalyzer (Agilent Technologies, Santa Clara, CA, USA). The Human Whole Genome OneArray^**®**^v6 (Phalanx Biotech Group, Taiwan) contains 32,679 DNA oligonucleotide probes, and each probe is a 60-mer designed in the sense direction. Among the probes, 31,741 probes correspond to the annotated genes in the RefSeq v51 and Ensembl v65 databases. In addition, 938 control probes are also included. The detailed descriptions of the gene array list are available from http://www.phalanx.com.tw/Products/HOA_Probe.php

### Microarray analysis

Fluorescent aRNA targets were prepared from 1 μg total RNA samples using the OneArray^**®**^ Amino Allyl aRNA Amplification kit (Phalanx Biotech Group, Taiwan) and Cy5 dyes (Amersham Pharmacia, Piscataway, NJ, USA). Fluorescent targets were hybridized to the Human Whole Genome OneArray^**®**^ with Phalanx hybridization buffer using the Phalanx Hybridization System. After 16 hr of hybridization at 50 °C, non-specific binding targets were washed away by three different washing steps (wash I 42 °C 5 min; wash II 42 °C, 5 min, 25 °C 5 min; wash III rinse 20 times), and the slides were dried by centrifugation and scanned by an Agilent G2505C scanner (Agilent Technologies, Santa Clara, CA, USA). The Cy5 fluorescence intensities of each spot were analyzed by GenePix 4.1 software (Molecular Devices). The signal intensity of each spot was loaded into the Rosetta Resolver System^**®**^ (Rosetta Biosoftware) to process data analysis. The error model of Rosetta Resolver System^**®**^ could remove both systematic and random errors from the data. We filtered out spots for which the flag was less than 0. Spots that passed the criteria were normalized by the 50% media scaling normalization method. The technically repeated data were tested by the Pearson correlation coefficient calculation to check the reproducibility (R value > 0.975). Normalized spot intensities were transformed to gene expression log_2_ ratios between the control and treatment groups. The spots with log_2_ ratio ≥ 1 or log_2_ ratio ≤ −1 and *P*-value < 0.05 were tested for further analysis.

### Quantitative RT-PCR

Total RNA was isolated by Trizol reagent (Invitrogen, Carlsbad, CA, USA) and reverse transcribed to produce cDNA using the Deoxy + HiSpec reverse transcriptase kit (Yeastern, Taipei, Taiwan) according to the manufacturer instructions. Q-PCR was analyzed with SYBR Green Master Mix (Applied Biosystems, Stockholm, Sweden) and subjected to quantitation in an Applied Biosystems LightCycler instrument. The primers used for PCR are given in Supplementary Table S5 online. The Q-PCR data were normalized to 18S cDNA levels.

## Additional Information

**How to cite this article**: Hsieh, T.-H. *et al.* A novel cell-penetrating peptide suppresses breast tumorigenesis by inhibiting β-catenin/LEF-1 signaling. *Sci. Rep.* **6**, 19156; doi: 10.1038/srep19156 (2016).

## Supplementary Material

Supplementary Information
