# Supplementary material for: A novel cell-penetrating peptide suppresses breast tumorigenesis by inhibiting β-catenin/LEF-1 signaling
Source: Sci Rep. 2016 Jan 11;6:19156. doi: 10.1038/srep19156 (PMC4707489; doi:10.1038/srep19156)
Supplement: Supplementary Information [file srep19156-s1.doc]

*Scientific Reports*

**Supplementary information**

**A novel cell-penetrating peptide suppresses breast tumorigenesis by inhibiting β-catenin/LEF-1 signaling**

**Tsung-Hua Hsieh^1,2,3^, Chia-Yi Hsu^5^, Cheng-Fang Tsai^5^, Chien-Chih Chiu^2^, Shih-Shin Liang^2^, Tsu-Nai Wang^2^, Po-Lin Kuo^2^, Cheng-Yu Long^1^ and Eing-Mei Tsai^1,2,3,4,5^***

^1^Department of Obstetrics and Gynecology, Kaohsiung Medical University Hospital, Kaohsiung Medical University, Kaohsiung, Taiwan

^2^Research Center for Environmental Medicine, Kaohsiung Medical University, Kaohsiung, Taiwan

^3^Center for Stem Cell Research, Kaohsiung Medical University, Kaohsiung, Taiwan

^4^Center for Infectious Disease and Cancer Research, Kaohsiung Medical University, Kaohsiung, Taiwan

^5^Graduate Institute of Medicine, College of Medicine, Kaohsiung Medical University, Kaohsiung, Taiwan

*Correspondence: Eing-Mei Tsai, MD, PhD, Tel.: 886-7-3121101 ext 6424, Fax: 886-7-311-2493, E-mail address: [tsaieing@yahoo.com](mailto:tsaieing@yahoo.com), Mailing address: No. 100, Zihyou 1st Rd., Sanmin District, Kaohsiung City 807, Taiwan

**
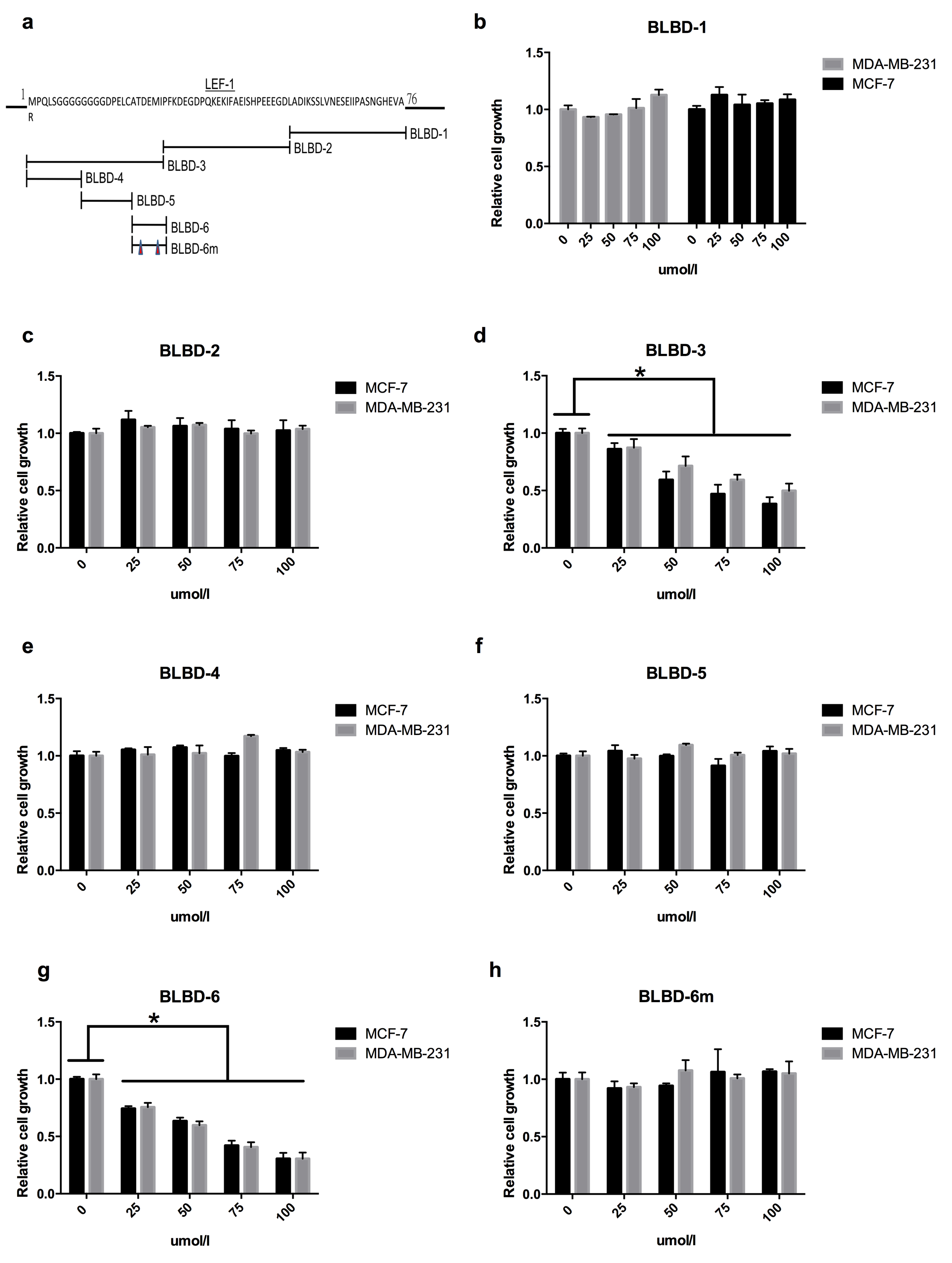
**

**Supplementary Figure S1. Dose-dependent effects of different BLBD fragments on cell growth.**

(a) The six BLBD peptides and the mutated sequence of the *β*-catenin/LEF-1 binding domain. (b-h) MCF-7 and MDA-MB-231 cells were transfected with the indicated doses of TAT-NLS-BLBD-1, -2, -3, -4, -5, -6, and -6m. Cell growth was analyzed by CCK-8 at 48 hr post-transfection. Data are the means ± SD of three experiments. **P* < 0.05 *vs*. untreated control; two-tailed Student’s *t* test.


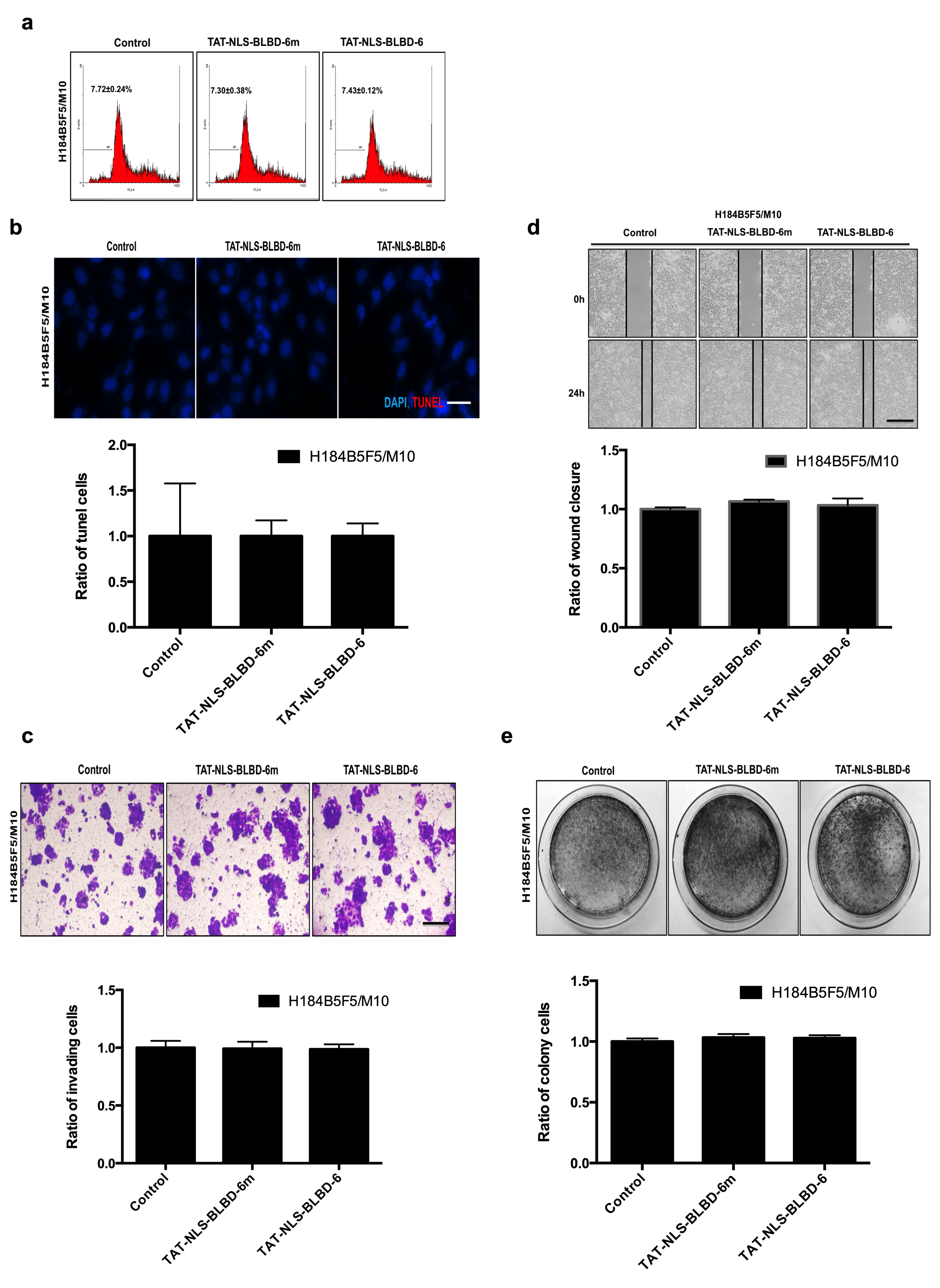


**Supplementary Figure S2.** **The phenotypic features of TAT-NLS-BLBD-6 in normal cell lines H184B5F5/M10.**

H184B5F5/M10 cells were transfected with control, 100 μmol/l TAT-NLS-BLBD-6 and TAT-NLS-BLBD-6m. (a) Cell cycle progression was analyzed by propidium iodide staining and flow cytometry. (b) Apoptosis was analyzed by the TUNEL assay. Arrows show apoptosis in the nucleolus. Motility was analyzed by the invasion assay (c) and wound-healing assay (d) at 48 hr post-transfection. Data are the means ± SD of three experiments. **P* < 0.05 *vs*. untreated control; two-tailed Student’s *t* test. (e) Cell proliferation was analyzed by the colony-formation assay at 14 day post-transfection. Scare bare=200 uM.

**
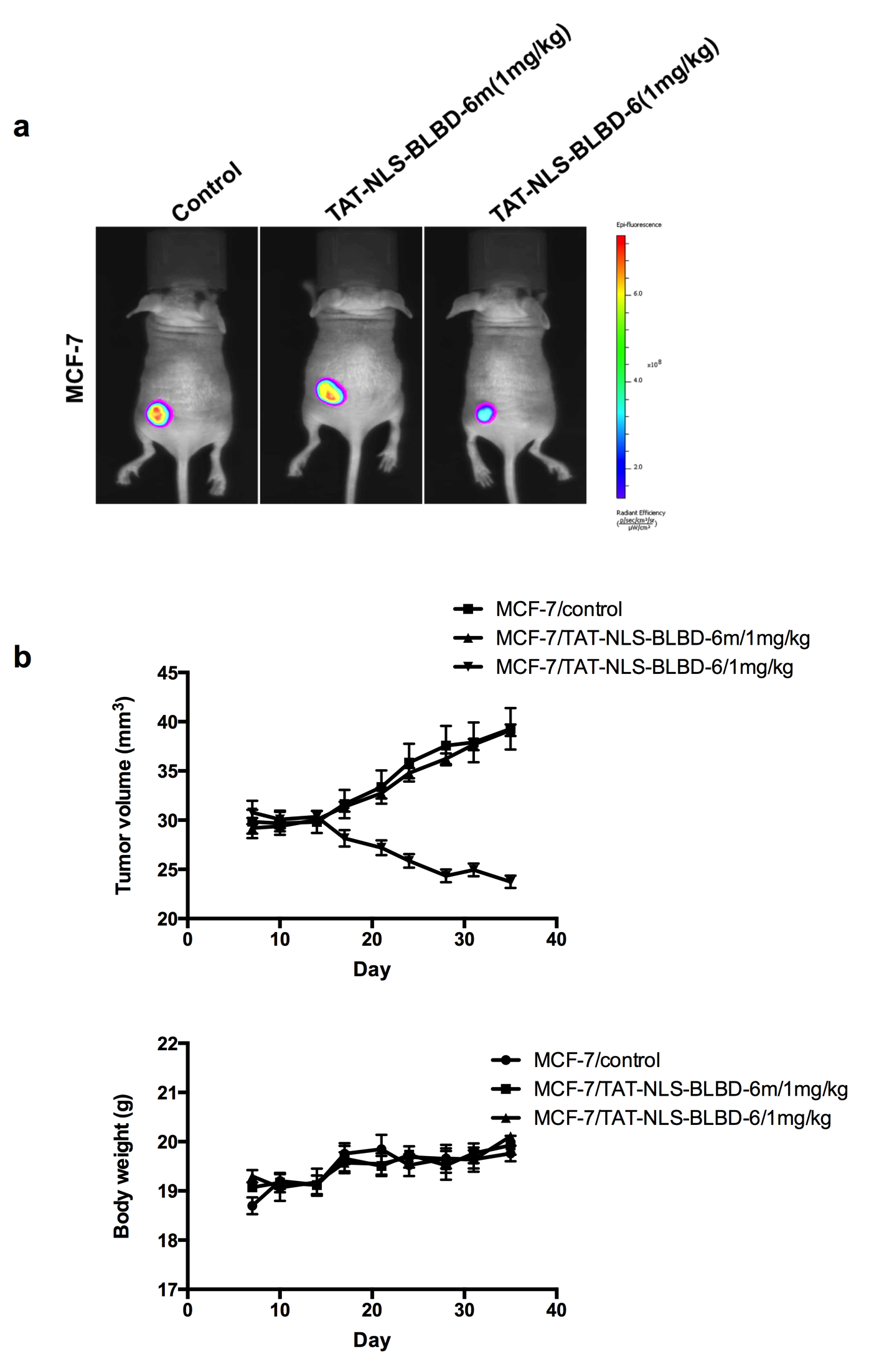
**

**Supplementary Figure S3. The tumor growth was decreased by TAT-NLS-BLBD-6 in nude mice.**

MCF-7-GFP cells were injected into the right side flanks of SCID nude mice (n = 5 per group). The control, 1 mg/kg TAT-NLS-BLBD-6 or TAT-NLS-BLBD-6m were injected into the tumor once every 2 days for 35 days. (a) Tumor GFP images were captured by whole body bioluminescence. (b) The tumor volumes and body weights of nude mice were calculated and recorded.


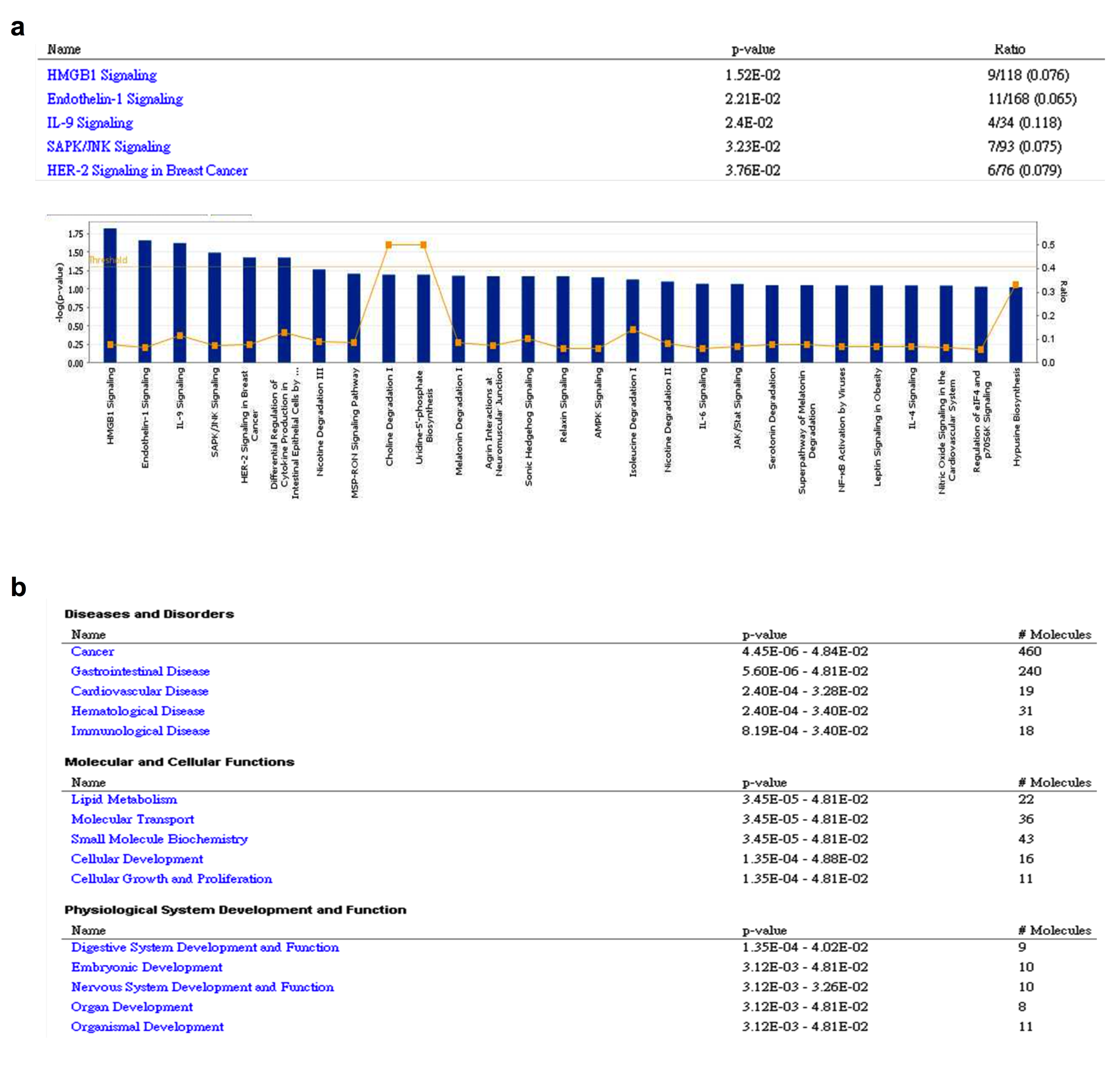


**Supplementary Figure S4. Pathway analysis of downstream target genes significantly down-regulated by TAT-NLS-BLBD-6 compared with TAT-NLS-BLBD-6m.**

(a) The associated pathways are HMGB1, endothelin-1, IL-9, SAPK/JNK, and HER2 signaling pathways. (b) Diseases and disorders, molecular and cellular functions, and physiological system development and function networks are shown. These gene pathways were analyzed using IPA software.


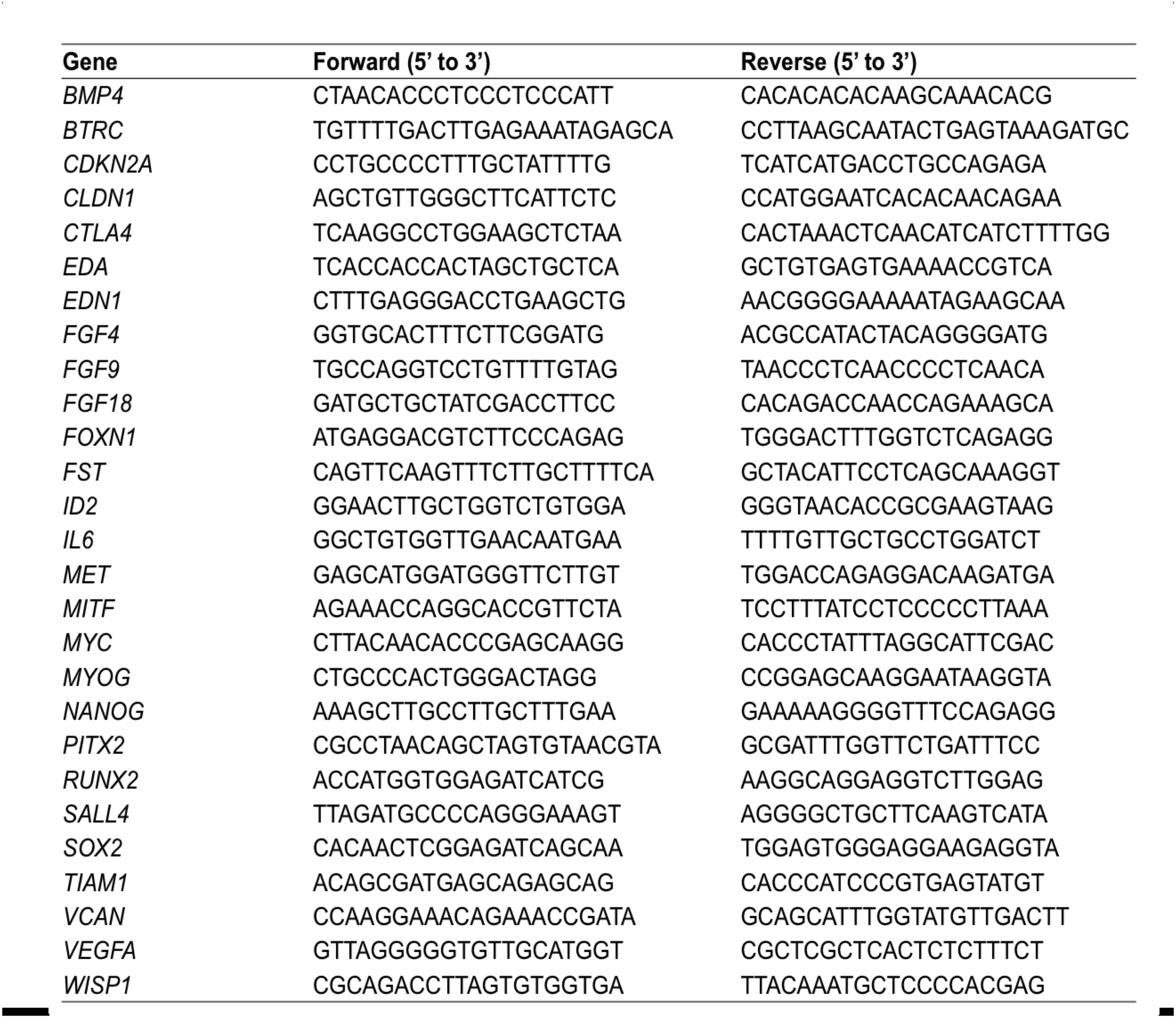


**Supplementary Figure S5. PCR primers for the 27 candidate genes down-regulated by TAT-NLS-BLBD-6.**
